# Some Hardware and Instrumentation Aspects of the Development of an Automation System for Jar Tests in Drinking Water Treatment

**DOI:** 10.3390/s17102305

**Published:** 2017-10-11

**Authors:** Antonio José Calderón, Isaías González

**Affiliations:** Department of Electrical Engineering, Electronics and Automation, University of Extremadura, Avenida de Elvas, s/n, 06006 Badajoz, Spain; ajcalde@unex.es

**Keywords:** jar test, automation, monitoring, instrumentation, water treatment

## Abstract

The so-called Jar Test (JT) plays a vital role in the drinking water and wastewater treatments for establishing the dosage of flocculants and coagulant. This test is a well-proved laboratory instrumental procedure performed by trained personnel. In this work, a completely novel system for the automation and monitoring of a JT devoted to drinking water treatment is presented. It has been implemented using an industrial programmable controller and sensors and instruments specifically selected for this purpose. Once the parameters of the test have been entered, the stages that compose the JT (stirring, coagulant addition, etc.) are sequentially performed without human intervention. Moreover, all the involved measurements from sensors are collected and made accessible for continuous monitoring of the process. By means of the proposed system, the JT procedure is conducted fully automatically and can be locally and remotely monitored in real-time. Furthermore, the developed system constitutes a portable laboratory that offers advantageous features like scalability and transportability. The proposed system is described focusing on hardware and instrumentation aspects, and successful results are reported.

## 1. Introduction

The access to water is one of the key challenges to solve towards the sustainable development of humankind. Scarcity of water is a huge problem, despite the efforts of governments and research institutions worldwide. One of the preeminent priorities of the 21st century consists on providing water to the world's population, while guaranteeing its availability for future needs [1]. Water is, in fact, a human right. A resolution of the United Nations in 2010 recognized the right to “safe and clean drinking water and sanitation as a human right” [2]. 

The environmental impact of the humankind activities are not limited to the greenhouse effect and global warming, but wastewater from industrial and urban processes also constitute a problem to address. In other words, the increasing Earth population and human actions imply pollution and contamination of water sources [3]. In addition, hazards associated to climate change like drought, floods or wind damage, can cause physical damage to water infrastructure and reduction of water availability [4]. Water Treatment Plants (WTPs) and water distribution networks are considered critical infrastructures, for instance, cyber-attacks on drinking water and wastewater facilities may cause cascading effects on public health, safety and economics [5].

Public health and environmental concerns have promoted the development of water treatment science and technology and the upgrading of treatment plants [6]. As a part of water resource management, drinking water and wastewater treatment play an essential role. Drinking water treatment makes water potable by removing contaminants present in the source water [6]. In the case of wastewaters from industrial activities, a chemical treatment is required before the wastewater can be discharged into the environment or into a biological treatment plant [7].

The more and more stringent regulations about water treatments have induced many advances in the automation of analytical procedures during the last years [8]. The design and application of monitoring and automation systems for water-related procedures is an active R&D domain. Some examples refer to water quality monitoring networks [9,10,11], control of water distribution networks [12], agricultural irrigation management [13,14,15], robotic platforms for water monitoring [16], urban drainage systems [17,18], and sensors for water assessment [19,20,21,22].

The presence in the water of many solid substances constitutes the most important and apparent part of the contamination. Colloidal particles are very small and stable, so he coagulation process consists on adding coagulant chemicals to neutralize their negative charges. Subsequently, the flocculation causes the formation of flocs as a result of collision and adhesion between those small destabilized particles, increasing their volume and weight so that they can decant. Within water treatments, the Jar Test (JT) is a well-known physical-chemical instrumental method to determine the optimal dosage of coagulant and flocculants. In fact, it is the standard procedure in water industry [23]. The American Society for Testing and Materials (ASTM) D2035 standard establishes a standard procedure for the JT [24]. JT is also used as reference and reliable procedure in the development of innovative treatment techniques like electrocoagulation [23], advanced oxidation processes [7], ceramic microfiltration of surface waters [25], or the design of software sensors based on neural networks or fuzzy logic [26].

Despite the importance of the JT procedure, most of its stages are mainly performed manually. Commercial apparatus offer solutions partially automated with the drawback of being closed, so modifications are subject to proprietary tools and specialized technicians to operate the system. On the other hand, the JT involves various analytical tasks and requires skilled personnel for accurate measurements and results, with a consequent bottleneck found where the analytics and related operations are performed by human operators. Therefore, the complete automation of such an important procedure constitutes a paramount step towards the generalization of its application and availability.

Deep analysis of the diverse parameters that affect the JT procedure is out of the scope of this paper, but the relevance of innovative automation and monitoring systems development has been clearly stated.

In this paper, the development of a novel automation and monitoring system for a JT devoted to drinking water treatment is presented. The key hardware and instrumentation aspects are described. The main contribution of the proposed system is that all the operations of the JT are performed automatically and autonomously. This kind of systems is currently non-existent either in the market or in the scientific literature. 

Among the available technology to automate processes, industrial Programmable Logic Controllers (PLCs) are the most suitable and versatile tools to carry out such task. A PLC is a real-time programmable electronic device hardened for harsh factory conditions. It is the most widely used controller in the industrial domain, used in different applications like renewable energy systems and smart grids [27], building automation [28], irrigation canals [13] and many others [29]. The control unit could have been implemented with by PC-based control software and a Data Acquisition card (DAQ) instead of a PLC, but we have chosen the industrial controller due to its well-known features of reliability, robustness and stable operation under the severe conditions of WTPs, and its advantageous small size and low power consumption. In addition, PLCs have built--in circuitry for collecting and conditioning the signals from the required sensors, using standardized ranges of digital and analog signals. The resolution and accuracy of measurements provided by PLCs is adequate for the reported development.

On the other hand, the ever-increasing development and application of Information and Communications Technologies (ICTs) have promoted the online access to information about the behavior of automated processes [30].

In the proposed approach, the controller is a PLC that senses and commands the different stages of the process by means of diverse sensors, actuators and instruments. In addition, a Human-Machine-Interface (HMI) implements a monitoring system function to continuously provide real-time information to the operator of the plant locally. This function is essential since a meaningful visualization of the process evolution contributes to an effective tracking and assessment. These components are connected through an Ethernet-based network and remote access to operational data is enabled using ICTs. To this aim, a web server hosted by the PLC allows online access to the JT results in real-time. 

The motivation of this work has been to implement an automated system to perform the JT in any WTP. Therefore, the developed prototype aims to implement effectively and in real-time the automation of a JT platform. In addition, the system has to be transportable and adaptive to any plant due to the fact that not all the plants have a chemical laboratory to carry out such a test every day. This portable laboratory is devoted to be applied in real WTP, but it can also act as a test bed or demonstrative facility for academic and/or R&D activities. Another important contribution of the proposal is the online availability of the relevant values of the JT. The target group of the paper is engineers and researchers, mainly in automation- and monitoring-related tasks, who may find such a paper as a valuable resource for the water treatment domain.

To the best of our knowledge, there are no automation systems to solve the JT problem under the framework proposed in this paper. The advantages of the presented proposal may be listed as follows:

- Stable operation due to the utilization of industrial equipment.
- Scalability and flexibility to add new instruments like sensors, actuators or other improvements using industrial open interfaces.
- Ability to monitor in real-time the process evolution, both in situ and remotely.
- Transportability and adaptability to perform the JT in different plants.

## 2. Materials and Methods 

In this section, we describe briefly the JT procedure as well as the components of the developed system.

### 2.1. Jar Test Description

The JT is a laboratory test for selecting and quantifying a treatment program for removal of suspended solids or oil from raw water or a dilute process or waste stream. Coagulation/flocculation units with differing chemical doses, mix speeds, and settling times are tested to estimate the minimum or ideal coagulant dosage required to achieve certain water quality goals. This method consists on a set of sequential steps that are conducted on a four- or six-place gang stirrer, which can be utilized to simulate mixing and settling conditions in a clarifier. Jars (beakers) with different treatment programs, or the same product at different dosages, are run side-by-side, and the results compared to an untreated jar, or one treated with the current program. Depending on the conditions of the WTP, the JT can be conducted with a periodicity that can vary from daily to weekly, monthly or seasonally. In this work, the implemented procedure is as follows:

- Fill the appropriate number of 1000 mL transparent jars with well-mixed test water.
- Mix the beakers at 100 rpm for 60 s (rapid speed). Continue mixing while coagulant addition is being carried out.
- Add, if necessary, pH corrector.
- Add increasing dosages of the coagulant to the beakers.
- Reduce the mixing to 20 rpm and continue the slow mix for 15 min. Continue mixing while flocculant addition is being carried out.
- Add increasing dosages of the flocculant to the beakers.
- Turn the mixer off and allow settling to occur for 20 min.
- After settling, extract the supernatant and filtered water for turbidity analysis.

**Figure 1:**
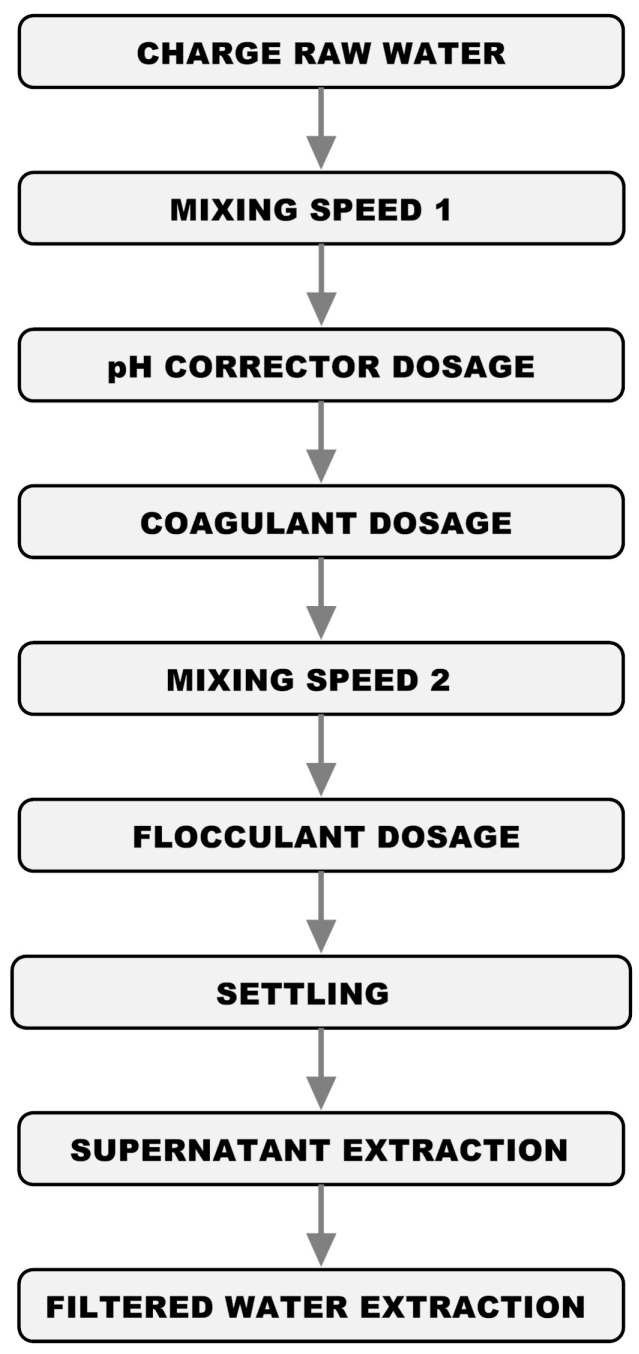
Flowchart of the JT procedure.

The flowchart depicted in Figure 1 shows the sequence of steps.

### 2.2. Automation and Monitoring Devices

The automation system comprises a Siemens S7-1200 PLC [31] equipped with analogue and digital Input/Output (I/O) modules. The models of these devices are now listed:

- CPU 1214C AC/DC/Rly.
- Two SM 1231 AI8 analogue input modules.
- SM 1232 AQ4 analogue output module.
- Two SM 1222 DQ16 digital output modules.
- HMI TP700 Comfort touch panel.

The chosen controller is a cost-effective PLC which belongs to Siemens low-end performance range with a modular and scalable design. It includes an integrated power source, a slot for a memory card, 14 digital inputs, 10 digital outputs, two analog inputs and an Ethernet communications port.

On the other hand, the HMI appliance is a high-end performance operator panel with a 7” Thin Film Transistor (TFT) color widescreen display and a processor running the embedded Windows CE operating system. It incorporates communications ports for Ethernet and Process Field Bus (PROFIBUS), three Universal Serial Bus (USB) connectors, two slots for memory cards, and supports Visual Basic scripts.

In regard to the configuration of the proposed system, the Siemens TIA Portal V13 software [32] has been used. It includes two different environments, one—Step 7 Professional—devoted to program the PLC control algorithm and the I/O management, and another one—WinCC—devoted to configure the monitoring and supervisory systems.

### 2.3. Instrumentation Devices

In order to measure the signals required to conduct the JT, various sensors have been applied and connected to the control unit. Besides, diverse actuators are needed to control the stages of the JT according to the PLC commands.

To begin with, water quality can be characterized by some physical parameters like temperature, pH or turbidity [10], being these two latter ones the main ones considered in the case of drinking water treatment. For pH sensing, a pH probe is required for each jar and all of them are connected to a digital pH meter. This pH meter provides an analog standardized 4–20 mA signal corresponding to each probe. The turbidity is sensed using a turbidity meter for continuous measurement compliant to the standard 7027 of the International Organization for Standardization (ISO) for determination of turbidity. 

The other variables to sense are the water level of the jars and the revolutions of the stirrer. The water level measurement is carried out using differential pressure sensors, whilst an optical encoder is used to register the revolutions of the stirrer. Table 1 lists the magnitudes involved in the JT operation and the corresponding sensors used to measure them, indicating model and manufacturer. These sensors are connected to the analog input modules of the PLC.

Concerning the actuators, various kinds of pumps are used jointly with valves. Namely, the dosing of coagulant/flocculant, the pH correction as well as the water supply and extraction is performed by means of dedicated pumps. In particular, a pump is used to supply raw water and another pump extracts water for the turbidity analyzer. Moreover, a DC motor acts as a stirrer to apply different mixing speeds to the water, whereas a magnetic stirrer is responsible of maintaining the pH corrector in proper conditions. These actuators are commanded using the analog output module of the PLC, except the pinch valves that are activated using the digital outputs. These devices are listed in Table 2 including the model and manufacturer.

Since the PLC uses standardized levels of digital and analog signals, most of the sensors and actuators are directly connected to its I/O modules without needing signal conditioning circuits. The only exception is the stirrer, as will be commented in Section 3.2.

Furthermore, the Jar Test JT40E equipment of the manufacturer OVAN [33] is used to implement the presented system. It consists of four jars of 1 liter each one, a DC motor for stirring and the corresponding four stainless steel rods. The prototype has been mounted into an industrial enclosure from the manufacturer Eldon [34].

## 3. Proposed System

**Figure 2:**
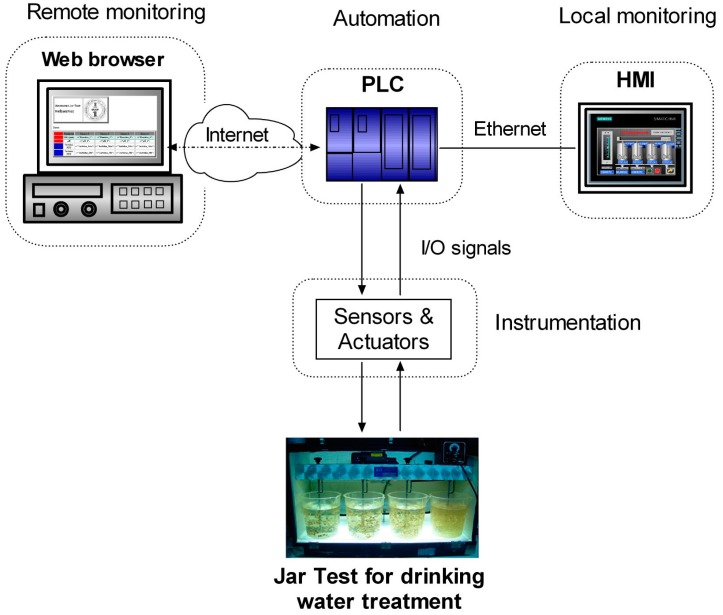
Block diagram of the developed system to automate and monitor the JT.

The proposed system can be considered as divided into four subsystems according to the implemented function, namely the automation subsystem, the instrumentation subsystem, the local monitoring subsystem and the remote monitoring subsystem. Figure 2 depicts the block diagram of the developed approach.

Despite the fact that the present paper is focused on the automation and monitoring system for the JT, it should be noted that this system is part of a larger project aiming at the design of an intelligent decision support system for the efficient management of WTPs. Specifically, the goal of this subproject was the automatic system design for the performance of the JT analysis, based on defining and dimensioning an automatic and portable system for carrying out the processes involved in the water coagulation/flocculation test (JT) from water quality indicators. To summarize, the main stages to complete the present work were the following:

- Determination of the procedures that can be automated.
- Defining methods for the automatic execution of all processes involved in the realization of the JT susceptible to automation.
- Dimensioning of the fluidic and mechanical subsystems, i.e., sizing of water circulation and transfer systems, controlled speed agitation, chemical doses, sample extraction, etc.
- Selection of the sensors to be used for the determination of the variables of interest in the analysis to be performed in the test: pH, turbidity.
- Designing an automatic system to perform the JT test in real time in a drinking WTP.
- Designing the prototype to be transportable and applicable at industrial level.
- Evaluation of the communication system used for the integration of the prototype in the complete monitoring system.
- Validation of the automatic system implemented to perform the JT in real time in a WTP.

### 3.1. Automation Subsystem

One of the most important parts of this work is the programming of the automation system, which will allow all the operations necessary to complete the JT cycle to be performed automatically. As aforementioned, the automation subsystem is based on a Siemens S7-1200 PLC. This controller incorporates built-in Organization Blocks (OBs), Functions (FCs) and Function Blocks (FBs). The OBs are executed cyclically. On the other hand, the developer can define function using FCs or FBs, the main difference is that the latter ones use data stored in a Data Block (DB). 

**Figure 3:**
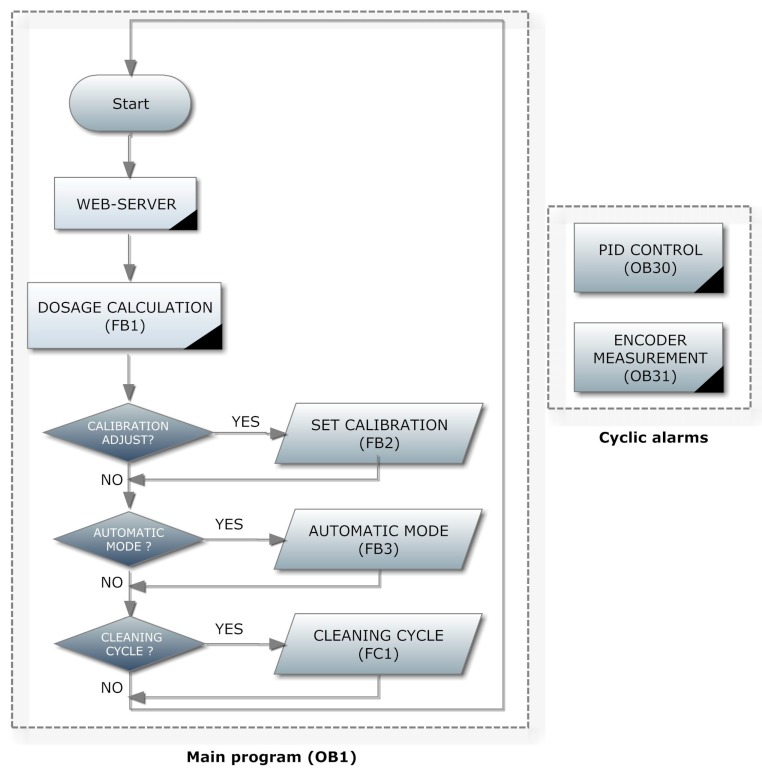
Flowchart of the program of the PLC.

Figure 3 shows the flowchart of the main structure of the control program (OB1) and of the cyclic alarms. The main program (OB1) will run cyclically while the PLC is in RUN mode, whereas the cyclic alarm blocks will run periodically at predefined intervals. The depicted stages are described in next paragraphs.

#### 3.1.1. Cyclic Alarms 

**Figure 4:**
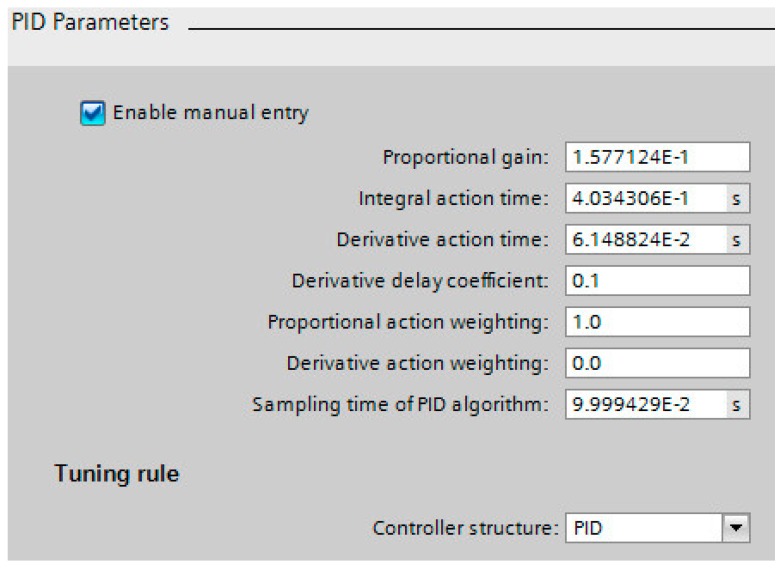
Configuration parameters of the PID controller.

Two periodic alarm blocks have been defined for the execution of a Proportional-Integral-Derivative (PID) control loop (OB30) and for the pulse reading of the encoder (OB31). The PID controller is used to regulate the speed of the stirrer, periodically executed each 100 ms. Before starting the controller, an initial optimization must be carried out so that the technological object itself adjusts the PID parameters, i.e., an auto-tune operation is applied. The obtained parameters for the PID controller are shown in Figure 4.

The periodic alarm OB31 is devoted to obtain the stirrer speed in rpm from the encoder measurement. To this aim, a High Speed Counter (HSC) of the PLC measures the frequency of the pulses provided by the encoder. Each 20 ms the calculations of the rpm are performed in such program block.

#### 3.1.2. Main Program

In this block the common tasks and calling to FBs and FCs are performed. The first stage is dedicated to a web server hosted by the PLC. The developed web server is detailed in the section devoted to the Remote Monitoring subsystem. After that, the calls to the used FBs and FCs are executed.

#### 3.1.3. Dosage Calculation

This block calculates the injection times in ms of each of the reagent pumps (coagulant and flocculant) in each of the test vessels. The procedure followed for calculating the injection time of each pump in each vessel is identical for the two pumps and the four test vessels. The calculation is made from the dose of coagulant or flocculant of each glass expressed in part per million (ppm) indicated in the recipe of the corresponding station. From this parameter and the calibrated flow value for each pump, the time to be dispensed of each pump in each vessel is obtained.

The dosing time (*DT*) of each reagent in ms is obtained from the equation:

(1) $$DT=\frac{FC}{FR}\times RD,$$

where *FC* is the flow, calibrated for each pump, *FR* is the flow rate introduced by the user and *RD* is the reagent dosage in mL. *RD* is obtained using the expression:

(2) $$RD=\frac{D}{AC}\times 1000,$$

where *D* is the dosage of the reagent in ppm, introduced by the user and *AC* is the actual concentration in ppm, obtained through:

(3) AC(ppm)=CC×1000×DF,

where *CC* is the concentration in g/l of the commercial solution, from which the solution used in the prototype is prepared. *DF* is the dilution factor applied to the stock dilution to obtain the solution used in the prototype. Both values are introduced by the user through the HMI.

#### 3.1.4. Set Calibration 

An important task to perform before the normal operation is the calibration of the different components. In this sense, the proposed system has been programmed to develop the calibration of the following devices: dosage pumps, pH pumps, pH meter, turbidity meter, and differential pressure sensors. These operations will be carried out at the time of the prototype set-up. In the case of the reagent pumps, with this operation the calibrated flow is obtained. This value will be used in the dosage calculations. 

#### 3.1.5. Automatic Mode

This block acquires special relevance within the control program since it is in charge of the automatic execution of the JT. In order to carry out the programming of this task the functional block has been structured in eight states, each of them solved with an FC function. This leads to a highly modular and highly flexible solution, since local modifications can be introduced in each phase easily without the need to change the block globally. The automatic mode, in fact, performs the sequence of steps depicted in Figure 1. The programmed states are:

- State 1: Filling beakers (FC1)
- State 2: pH correction (FC2)
- State 3: Mixing at (high) speed 1 (FC4)
- State 4: Coagulant dosage (FC3)
- State 5: Mixing at (low) speed 2 (FC4)
- State 6: Flocculant dosage (FC6)
- State 7: Settling time (FC4)
- State 8a: Supernatant extraction (FC5)
- State 8b: Filtered water Extraction (FC8)

The last stage of the automatic execution of the JT corresponds to the extraction of supernatant samples to perform the turbidity analysis. This extraction will be carried out in two phases. In the first, the supernatant extracted in each beaker is taken directly to the turbidity meter. In the second phase, the extracted supernatant is passed through a filter before being taken to the turbidity analyzer. Both phases are identically repeated for all the vessels.

#### 3.1.6. Cleaning Cycle

A cleaning function has been implemented in order to automatically clean the tumblers once finished the JT. This block implements the necessary programming to carry out the following actions:

- Sequential filling of vessels 1–4.
- Agitation throughout the cleaning process, once the vessels have been filled.
- Sequential extraction of decanted water in the vessels.
- Sequential extraction of filtered water in the vessels.
- Simultaneous emptying of the vessels.

### 3.2. Instrumentation Subsystem

The components of this subsystem have been described in the Section 2.3, however, hereafter some design considerations about them are discussed. When designing the system, an important question was considered—how to develop a compact and portable laboratory. As aforesaid, a noteworthy fact is that the presented solution offers the feature of being scalable and flexible. In contrast to commercial approaches that use closed configurations, the reported sensors and actuators manage standardized I/O ranges and, thusly, can be easily repaired, substituted and/or expanded. It should be taken into account that the selected instrumentation equipment provides the same level of accuracy that those used nowadays in laboratories. This work is expected to become a first step to improve the reliability and repeatability of the JT.

Concerning the sensors, a pH probe is inserted into each jar to measure the pH value, and the four probes are connected to the digital pH meter to be processed. After that, the pH meter is linked to the PLC via 4–20 mA signals to consider the measurement in the control program. To measure the water level inside the jars, differential pressure sensors are applied, so they provide an analog signal in the range 4–20 mA proportional to the pressure in the jar. The high sensibility of these sensors makes them suitable for an accurate and continuous measurement of the water volume. As far as turbidity is concerned, the water is extracted and passed through the turbidity meter both directly and another through a filter. This way, the turbidity of both the decanted water and the filtered one is measured and registered.

**Figure 5:**
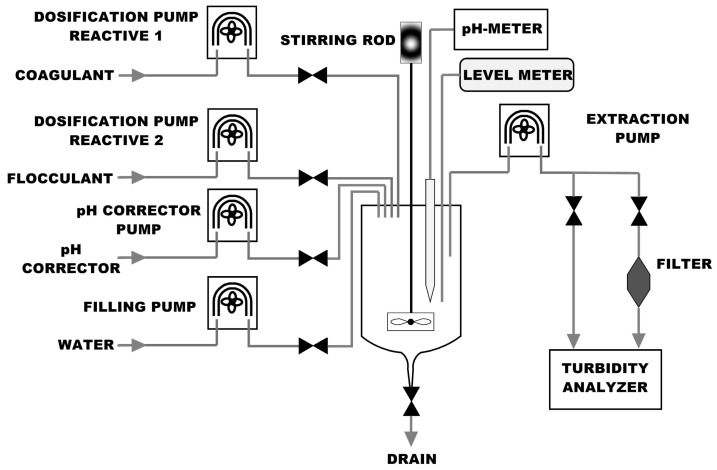
Fluidic diagram of the proposed solution including the sensors and actuators.

Regarding the actuators, in order to the PLC is able to take control of the DC motor, it is necessary to insert as a pre-actuator a DC regulator (Cebek board) whose setpoint is supplied through an analog output. This output is, in turn, the output of the PID control loop which has been implemented in the PLC for speed control of the stirring motor. The DC regulator is necessary to be able to power the motor properly, since the output of the analog module of the PLC provides a voltage between 0 and 10 V, and a voltage between 0 and 24 V is required for the motor. The 0–10 V output signal delivered by the analog module will be used as the control input to the board. The feedback signal is provided by the incremental encoder. The pinch valves apply a pinching effect to control the fluid flow within a flexible tube, only the inner face of the tube remains in contact with the fluid. These valves has been chosen since they minimize the apparition of turbulences and dead volumes in comparison with other types of valves like diaphragm valves for instance, making them ideal for applications with a high level of hygiene of the fluid. Apart from the mechanical stirrer for water mixing, a magnetic stirrer with heating function is required to maintain the pH corrector in optimal conditions to be used in the JT. For a better comprehension of the proposed solution, Figure 5 outlines the fluidic diagram from the standpoint of each beaker including all the sensors and actuators.

### 3.3. Local Monitoring Subsystem

As it is evident, once the process of the JT is fully automated, a human operator of the WTP needs to access locally the real-time data of the procedure in order to verify the correct evolution. In addition, numerous tasks can also be commanded by the operator to define the initial parameters of the test, as well as maintenance tasks like calibrations or cleanings. To satisfy these requirements, in order to provide the PLC with the ability to interact with a user, the HMI is responsible of displaying the values of the parameters and allowing the operator’s intervention. 

The information gathered from the PLC is provided by the HMI to the operator continuously including numerical data and illustrative synoptic diagrams, refreshed in real-time. The developed interface comprises numerous screens to offer information in an intuitive and easy-to-use layout, providing a user-friendly environment. 

**Figure 6:**
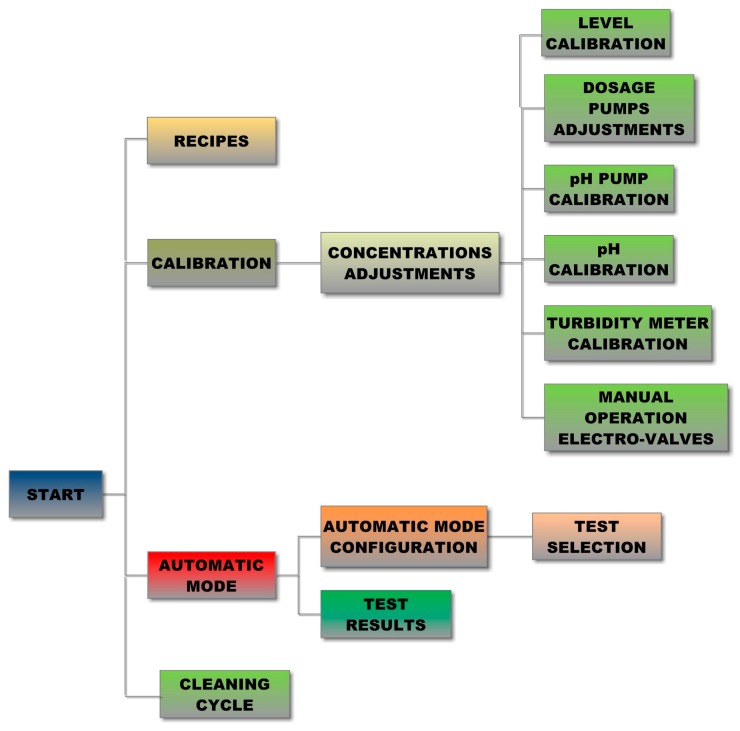
Navigation tree of the HMI.

User-configurable ability delivers some choices to command diverse operations or modify parameters. Figure 6 outlines the navigation tree of the developed HMI according to the different options that the user can select. 

Specifically, from the initial screen, the following operations can be managed:

- Recipes: To select the default parameters assigned to the tests of each WTP.
- Calibration: To adjust the parameters of the dosage pumps (flocculant and coagulant), pH pump calibration, pH meter calibration, turbidity meter calibration, calibration of the level meter and manual activation/deactivation of extraction and discharge.
- Automatic Mode: Starts the automatic operation of the JT.
- Cleaning cycle: For filling or emptying of the beakers or performing a cleaning cycle after each test.

**Figure 7:**
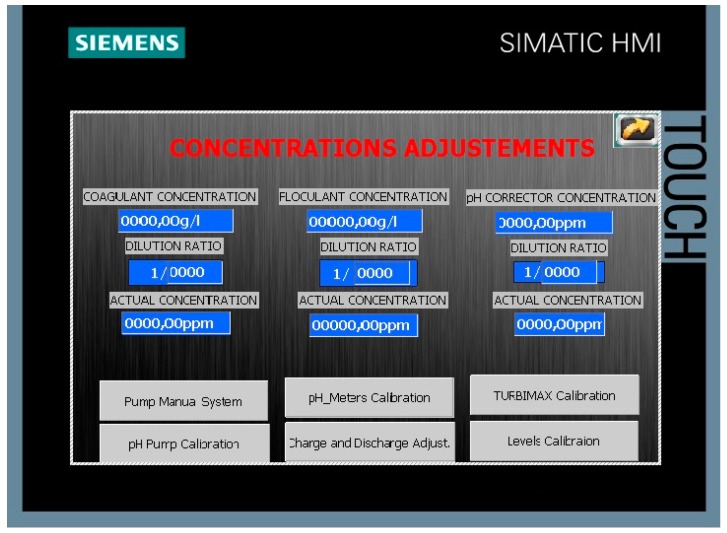
Screen to modify concentrations in the HMI.

**Figure 8:**
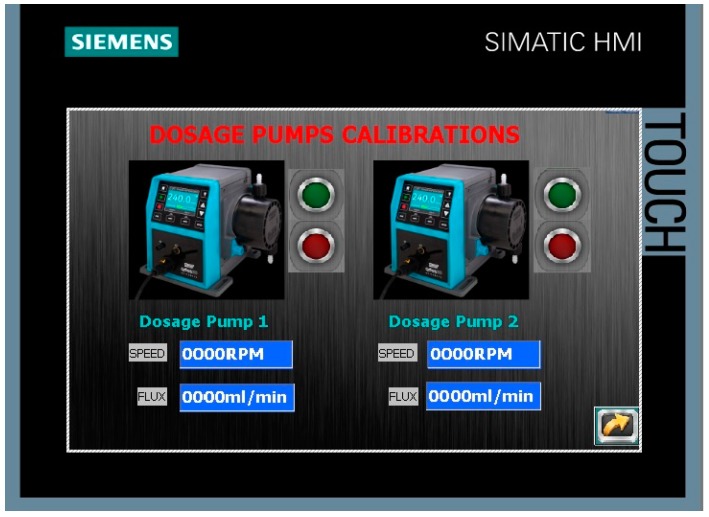
Screen to calibrate the dosage pumps in the HMI.

The most illustrative screens of the designed HMI are now commented. Figure 7 shows the screen devoted to enter the values of parameters like the concentration of coagulant, flocculant, and pH corrector, used to determine the corresponding dosages. Regarding the calibration options, for instance, the screen devoted to manage the calibration of the dosage pumps is observed in Figure 8. The user must enter the mixing speed and the flow for each one of the pumps, and press the green button to start the calibration process, which is executed automatically. 

### 3.4. Remote Monitoring Subsystem

The remote monitoring ability constitutes an essential functionality in the proposed system, so a web server has been implemented. This server allows access to the data of interest obtained after the realization of the tests. The remote user accesses online to the values hosted by the web server using a common web browser and a Transmission Control Protocol/Internet Protocol (TCP/IP) connection. This feature enables a flexible monitoring of the test that may promote the collaboration between distant partners/operators under a decentralized framework of the WTP operation.

Data gathering between the PLC and the web server is directly conducted due to the fact that such web server is hosted in the own PLC. This way, the Hyper Text Markup Language (HTML) code processes the information of the PLC memory and offers it according to the established configuration. Once the CPU is in RUN mode, the user-defined web page can be opened using a web browser. Therefore, this web server allows customized designs so the developer is able to identify the most relevant parameters of the process, the JT in this case, and to implement the interface in an intuitive way according to the expected utilization. In the proposed approach, we can assert that the developed web page fulfills entirely the requirements for a seamless remote monitoring of the JT results. In fact, a simple but effective application has been implemented through the web server. The resulting appearance of the user web page is shown in the Results section. 

## 4. Results

In this section, in order to validate the developed system, the experimental results are now exposed. These results demonstrate the feasibility and suitability of such a system to sense, automate and monitor the JT procedure. The experimentation has been conducted in the Automation and Industrial Informatics Laboratory of the Electrical Engineering, Electronics and Automation Department of the University of Extremadura (Spain).

### 4.1. Implemented System

**Figure 9:**
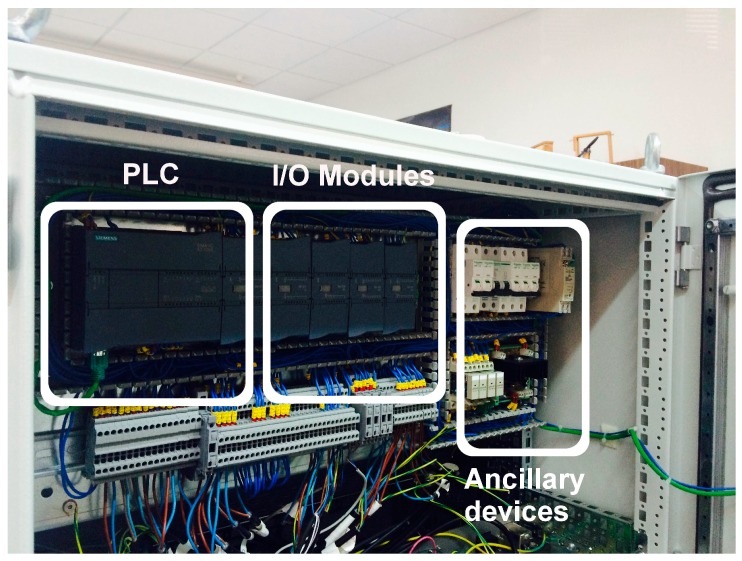
Automation subsystem.

**Figure 10:**
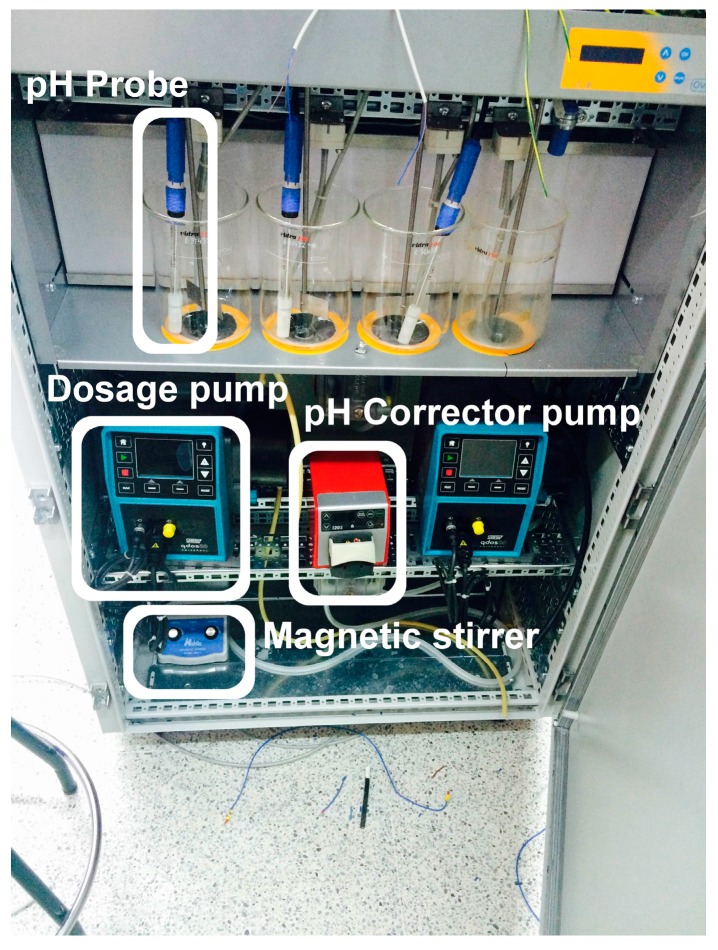
Front view of the prototype.

**Figure 11:**
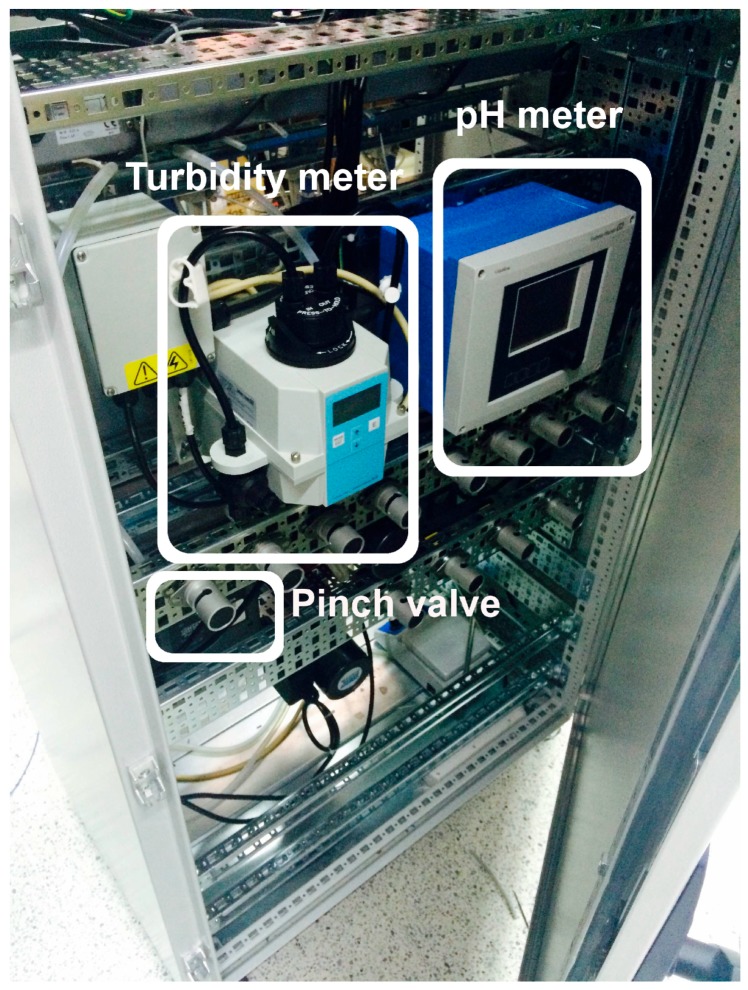
Rear view of the prototype.

Figure 9 shows a photograph of the already implemented automation subsystem, where the PLC with the additional modules (analog and digital I/Os) and the ancillary devices (connectors, relays, etc.) can be appreciated. The front view of the prototype including the four jars, the pH probes, the dosage pumps, the pH correction pump, and the magnetic stirrer can be seen in Figure 10. Finally, the rear view of the system can be observed in Figure 11, where the digital pH meter, the turbidity meter and the electro valves are clearly grasped.

### 4.2. Evaluation and Test Results

A noteworthy feature is that the results of the JT must not depend on the automation system, i.e., their values and evolution should be independent of the human or automated operator that carries out the tasks. However, the availability of the obtained results in real-time does provide crucial information for the operator and for the higher supervisory level of the entire WTP in order to perform the management and execution of the water treatment.

Before describing the achieved results, it is interesting to give some background about the criteria used for evaluating the quality of drinking water. According to the World Health Organization (WHO), low turbidity (measured in Nephelometric Turbidity Units, NTUs) in drinking water is an indicator of pathogen removal and of safety [35]. Water is visibly cloudy at 4 NTU and above, so the aesthetic acceptability of drinking water requires lower levels [35]. As a guide, “crystal-clear” water has a turbidity below 1 NTU [35]. About the pH value, the WHO indicates that the pH of most drinking water lies within the range 6.5–8.5 [36].

**Figure 12:**
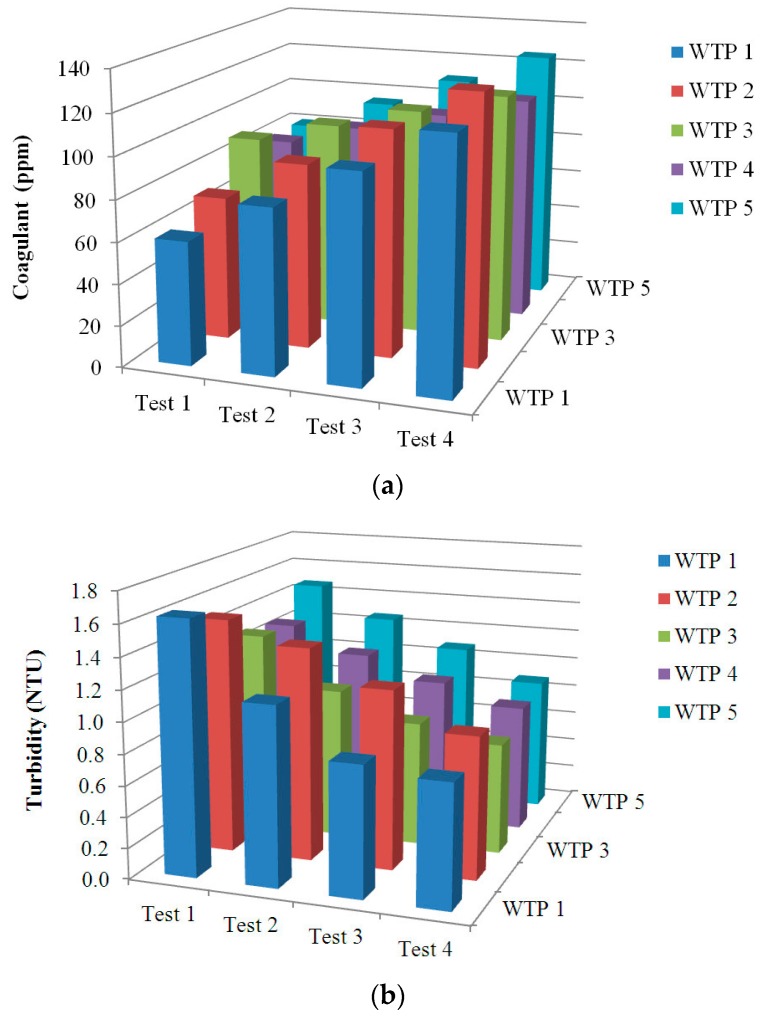
Three-dimensional graph of results of analyses performed: (**a**) Coagulant dosage; (**b**) Turbidity of filtered water.

For the validation of the prototype, a set of JTs have been carried out using raw water from five different WTPs placed in the region of Extremadura, Spain. For the raw water of every WTP the JT provides four groups of values corresponding to each vessel. In other words, each vessel has a coagulant dosage different to the rest, so a single test is performed in each one. This way, the Test 1 is performed in the first vessel and so on. The dosages are increased from the first to the last vessel in order to find the minimum amount required to achieve a turbidity below 1 NTU. Moreover, different coagulant dosages have been used for each JT in order to emulate the operation in real WTPs, where the dosages are proposed as a result of the experience in the treatment of the raw water that arrives. Figure 12 shows a three-dimensional representation of the data corresponding to the coagulant dosage, in ppm (Figure 12a), and the measured turbidity, in NTU, of the filtered water (Figure 12b). As can be observed, the obtained results show values within the expected ranges. In addition, on the view of the values, the selected optimal dosage for each WTP can be selected according to the aforementioned criteria.

**Figure 13:**
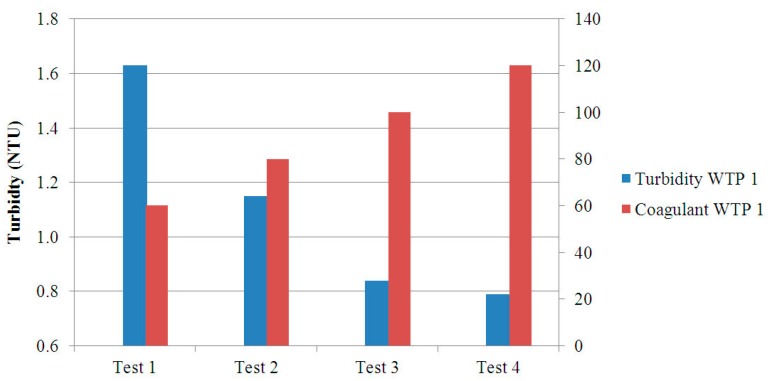
Turbidity and coagulant dosage values for each vessel in the JT of the WTP 1.

A detailed view of the JT results for the water from the WTP numbered as 1 is represented in Figure 13 by means of a bar chart. The values of turbidity of the filtered water and the associated coagulant dosage for each test are shown. The increase of coagulant dosage between tests is 20 ppm. On the other hand, the turbidity levels show a decreasing tendency as the coagulant dosage increases. On the view of these results, the dosage to achieve an optimal drinking water treatment corresponds to the Test 3, 100 ppm, since this is the minor amount that allows a turbidity level under 1 NTU.

Evidently, a number of trials have been carried out to verify the correct interconnection of the components and the automation of each step. The partial results obtained during these trials are not meaningful so they are not reported. Once the prototype was fully built, for each JT, the developers have supplied the raw water for each jar and have introduced the required parameters (coagulant dosage, mixing speed setpoint and so on) by means of the HMI. The prototype has operated autonomously performing sequentially the stages that compose the JT and providing the displayed successful results. As indicated at the beginning of the section, the JT results do not depend on the nature of the operator, and taking into account that the obtained values are within the expected ranges, the proposed system has been experimentally validated. As mentioned in Section 3, this prototype is part of a larger project supported by a company which is its owner and final user. The results have been subsequently validated by the company performing the same tests in its laboratories.

**Figure 14:**
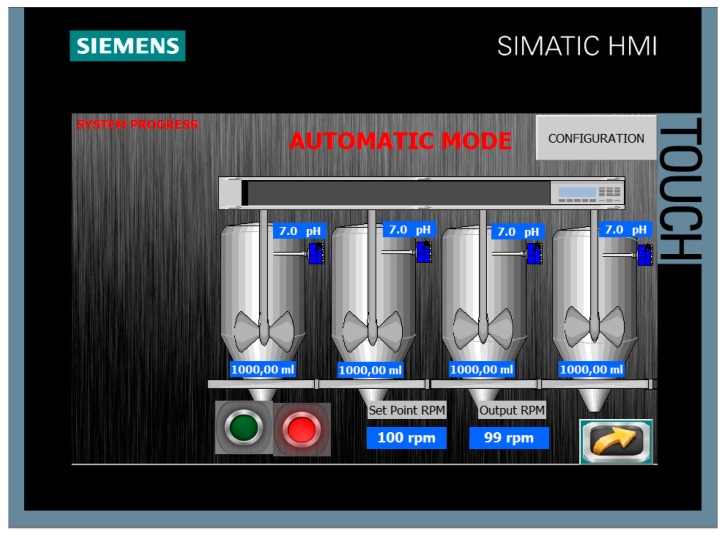
Screen of the HMI to illustrate the evolution of the JT.

**Figure 15:**
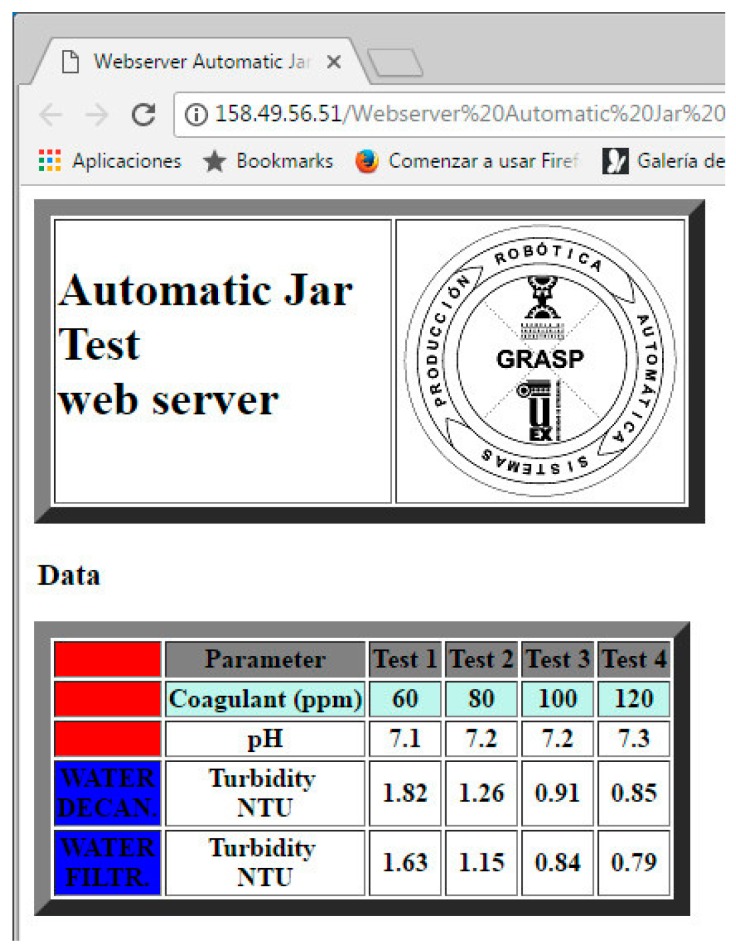
Web page for remote monitoring of the JT results.

To reflect the effective behavior of the prototype, the results corresponding to the JT carried out with the water from the WTP number 1 are shown. A screenshot of the HMI to illustrate the evolution of the JT is observed in Figure 14. It has been taken at the beginning of the process, so the pH values and volumes are the same for all the vessels. The web page provided to the remote user by the web server is shown in Figure 15. As can be observed, a table contains the results for each one of the jars, namely, the coagulant dosage, the pH, and the turbidity for both decanted water and filtered water.

## 5. Conclusions

This paper has presented a completely novel system for monitoring and automation of a JT devoted to drinking water treatment. The most outstanding contribution of the proposed system is that all the operations of the JT are performed automatically and autonomously. This kind of system is non-existent either in the market or in the scientific literature. 

A set of sensors measure the pH and the turbidity of raw and filtered water. Actuators like stirrers, pinch valves and pumps are governed by a Siemens PLC to determine the optimal dose of coagulant/flocculants. To carry out local monitoring, a HMI device is responsible of feeding the operator with information about the JT operation in real-time. On the other hand, taking advantage of ICT resources and a PLC-hosted web server, a remote operator can access online to the JT results. Despite the automation of the process, nowadays, the human intervention is still required to introduce relevant parameters like dosages, mixing speeds, etc. Moreover, maintenance tasks (cleaning and calibration) are directly performed without stoppage for disassembling the system.

The reported results prove the feasibility of the developed portable laboratory, which offers features like scalability and transportability, fulfilling the established requirements.

It is worth mentioning that one of the goals of this work has been the abstraction of the results regardless of which they are achieved under the traditional method in the laboratory or using the proposed automatic prototype.

Within a R&D context application, modifications in the control algorithm implemented by the PLC can be easily performed, enabling the study of different strategies. Also, the evaluation of diverse sensors and instrumentation technologies can be carried out.

Data storage is a useful functionality to carry out the surveillance of the operations, indeed, prognostics and preventive maintenance tasks can be based on such data. In this sense, to improve the presented system, future guidelines include the utilization of cloud-enabled storage of the sensed data. Due to the fact that WTP are critical infrastructures, cyber security issues are also being studied. A complementary study may be to analyse the errors and uncertainties associated to instrumentation. 

## Figures and Tables

**Table 1:** Summary of magnitudes and sensors for the JT.

Magnitude	Sensor
pH	pH meter Endress Hauser CUE21-A1A Liquiline CM444
	pH probes CPS11D-7AA21
Turbidity	Endress Hauser Turbimax CUE21
Level (differential pressure)	PSE550-28 SMC
Revolutions/angular speed	US DIGITAL E5 Optical Kit Encoder

**Table 2:** Summary of actuators and devices for the JT.

Actuator Function	Device
Pumps for dosage	Watson Marlow Qdos
Pinch valve	Wedge solenoid valve 16.003.125 Series
Pump for pH correction	Watson Marlow 120S
Pump for supply and extraction	Watson Marlow Alitea
Stirrer	DC motor
Magnetic stirrer	Nahita magnetic stirrer

## Abbreviations

The following abbreviations are used in this manuscript:

DAQ	Data Acquisition Card
DB	Data Block
FB	Function Block
GUI	Graphical User Interface
HMI	Human Machine Interface
HTML	Hyper Text Markup Language
HSC	High Speed Counter
ICTs	Information and Communications Technologies
IP	Internet Protocol
I/O	Input/Output
ISO	International Organization for Standardization
JT	Jar Test
NTU	Nephelometric Turbidity Unit
OB	Organization Block
PC	Personal Computer
PID	Proportional-Integral-Derivative
PLC	Programmable Logic Controller
PROFIBUS	Process Field Bus
TCP	Transmission Control Protocol
TFT	Thin Film Transistor
USB	Universal Serial Bus
WHO	World Health Organization
WTP	Water Treatment Plant
